# Hypoxia exposure alleviates impaired muscular metabolism, glucose tolerance, and aerobic capacity in apelin‐knockout mice

**DOI:** 10.1002/2211-5463.12587

**Published:** 2019-01-23

**Authors:** Shiyi He, Junping Li, Jianxiong Wang, Ying Zhang

**Affiliations:** ^1^ School of Sport Science Beijing Sport University China; ^2^ Faculty of Health, Engineering and Sciences University of Southern Queensland Toowoomba Australia

**Keywords:** apelin, diabetes, hypoxia, metabolic disorder

## Abstract

High altitude hypoxia adaptation can improve glucose tolerance in people with metabolic syndrome and type 2 diabetes (T2D). Apelin is an endogenous ligand of the G protein‐coupled receptor APJ and has possible roles in energy metabolism. Apelin‐null mice have been reported to exhibit impaired insulin sensitivity, which can be reversed by supplementation of exogenous apelin. Here, we examined the effects of 4 weeks’ intermittent hypoxia exposure on physiological and biochemical variables in apelin knockout (KO) mice. Apelin KO mice exhibited decreased expression of substrate metabolism‐associated genes/proteins, impaired glucose tolerance, and reduced exercise capacity compared to wild‐type mice, and all of these effects were rescued by hypoxia. These findings suggest that hypoxia intervention may possibly be able to alleviate metabolic conditions caused by genetic defects.

**Abbreviations**
AMPKα: AMP‐activated protein kinase α
AUC: area under the curve
COX4‐2/*Cox4‐2*: cytochrome *c* oxidase subunit 4 isoform 2
ERRα/*Esrra*: estrogen‐related receptor α
*Gbe1*: 1,4‐α‐glucan branching enzyme 1
GLUT4: glucose transporter 4
GTT: glucose tolerance test
HIF1: hypoxia‐inducible factor 1
*Hk2*: hexokinase 2
ITT: insulin tolerance test
KO: knockout
NRF1/*Nrf1*: nuclear respiratory factor 1
*Pfkm*: muscular phosphofructokinase
*Phka1*: phosphorylase kinase regulatory subunit α 1
PPARα/*Ppara*: peroxisome proliferator‐activated receptor α
*Rn18s*: 18S ribosomal RNA
*Slc2a4*: glucose transporter type 4
T2D: type 2 diabetes
TFAM/*Tfam*: mitochondrial transcription factor A
UCP3/*Ucp3*: uncoupling protein 3
${\dot{V}}_{O_{2}\max}$: maximal oxygen uptake

People living at high altitude usually have lower blood glucose levels or lower incidence of type 2 diabetes (T2D) compared with plain inhabitants 1. High altitude hypoxia adaptation can improve glucose tolerance in people with metabolic syndrome 2 and T2D 3. Under laboratory conditions, 4 weeks’ intermittent hypobaric hypoxia exposure has been demonstrated to improve the impaired glucose tolerance in diabetic rats 4, and decrease fasting blood glucose and insulin resistance in fructose‐induced metabolic syndrome rats 5. Normobaric hypoxia training led to loss of body mass or amended metabolic risk factors in people who were overweight or obese 6. These findings imply that hypoxia may be efficient for treating the metabolic abnormalities in the body.

Apelin is an endogenous ligand of the G protein‐coupled receptor APJ 7. It widely expresses in mammalain tissues and is associated with functional effects in the central and peripheral regulation of the cardiovascular system, water and food intake and immune function 8. Previous studies have also reported possible roles of apelin in energy metabolism. Plasma apelin concentration is increased in people with obesity 9, T2D patients 10 and hyperinsulinemic obese mice 11. The apelin–APJ system has become a therapeutic target in the treatment of diabetes and its complications 12. Some studies have shown that exogenous apelin stimulates glucose utilization in skeletal muscle, the major site of glycemic control and metabolic homeostasis 13, in both normal and insulin‐resistant mice 14, 15, and increases fatty acid oxidation, mitochondrial oxidative capacity and biogenesis in skeletal muscle of insulin‐resistant mice 16. Notably, some negative outcomes caused by apelin deficiency have been indicated in apelin knockout (KO) mouse models. For example, a study found that the apelin‐null mice had impaired insulin sensitivity, which could be reversed by supplementation of exogenous apelin 14. In addition, without a functional apelin–APJ signaling pathway, apelin KO mice exhibited a decrease in exercise capacity in a previous study 17.

As reviewed above, hypoxic exposure may have effects on improving glucose tolerance and insulin sensitivity; however, there has been no report regarding the application of hypoxic exposure to treat apelin KO mice in the literature. Therefore, we aimed to test the effects of 4 weeks’ intermittent hypoxia exposure on physiological and biochemical variables in apelin KO mice, which are closely associated with metabolic disorder. The outcomes of the current study provides molecular evidence for hypoxia exposure affecting muscular energy metabolism, glucose and insulin tolerance, and aerobic capacity (measured by maximal oxygen uptake, ${\dot{V}}_{O_{2}\max}$) in apelin KO mice. We hypothesized that the apelin KO mice would exhibit impaired glucose and insulin tolerance, as well as low exercise capacity. The findings indicated that these effects would be reversed by hypoxia exposure, which might be achieved, at least in part, through the improvement of muscular substrate metabolism in apelin KO mice.

## Materials and methods

### Animal care

The experimental procedure was conducted in accordance with the Guide for the Care and Use of Laboratory Animals of the Beijing Sport University. The protocol was approved by the Animal Care and Use Committee of Beijing Sport University, China.

### Animals

Apelin^−/−^ (also known as apln^−/−^) mice (B6; 129S5‐Apln^tm1Lex^/Mmucd, RRID: MMRRC_032151‐UCD) were purchased from Mutant Mouse Resource & Research Centers (MMRRC; davis, CA, USA) and bred in‐house on a C57BL/6J genetic background. Male apelin^−/−^ mice and apelin^+/+^ littermates (20 ± 2 g, 8 weeks old), herein referred to as apelin KO and wild‐type (WT) mice, respectively, were housed in a temperature‐ and light‐controlled environment (20–25 °C and 12‐h light–dark cycle). Food and water were supplied *ad libitum*. Apelin KO and WT mice were randomly allocated into four groups: WT–Normoxia, WT–Hypoxia, KO–Normoxia, and KO–Hypoxia, with 18 mice in each group. Nine mice in each group were used for the glucose tolerance test (GTT), insulin tolerance test (ITT) and ${\dot{V}}_{O_{2}\max}$ test, while the remaining mice were used for plasma and tissue collection.

### Hypoxia exposure

The hypoxia exposure was achieved by placing the mice in a normobaric chamber (210 cm long, 200 cm wide, and 200 cm high). The chamber was infused with hypoxic air through an air compressor and a nitrogen synthesizing machine, which could reduce the oxygen concentration in the chamber to 13.3% (at a simulated altitude of ~ 3500 m). The oxygen concentration in the chamber was monitored throughout the experimental period with an oxygen sensor. The hypoxic treatment was performed for 8 h per day during the daytime for a total of 4 weeks.

### PCR analysis

Apelin disruption was confirmed by PCR genotyping. Tail genomic DNA was amplified with specific primers. The amplification products of the WT (176 bp) and mutant (331 bp) apelin alleles were separated on a 1.5% agarose gel for visualization using electrophoresis according to the protocol from the MMRRC website.

### ${\dot{V}}_{O_{2}\max}$ test

${\dot{V}}_{O_{2}\max}$ has been referred to the gold standard of aerobic fitness 18. To avoid acute effects of the last hypoxia session, the mice of the WT–Hypoxia and KO–Hypoxia groups rested for 48 h under normoxia with food and water accessible prior to ${\dot{V}}_{O_{2}\max}$ determination. ${\dot{V}}_{O_{2}\max}$ of all mice was determined using a slightly modified incremental treadmill test 19 with a Comprehensive Lab Animal Monitoring System (Columbus Instruments International Corp., Columbus, OH, USA). Briefly, the treadmill was set to an incline of 5° and a starting speed of 10 m·min^−1^ for 10 min. After this initial phase, the speed was increased by 3 m·min^−1^ every 3 min until the mouse spent > 10 s on the shock grid without attempting to continue running 20. Once exhaustion was reached, the power of the shock grid was turned off, and ${\dot{V}}_{O_{2}\max}$ was calculated.

### GTTs and ITTs

After the ${\dot{V}}_{O_{2}\max}$ test, the mice rested for 48 h and had access to food and water. A GTT was conducted after a further 16 h of fasting. The mice received intraperitoneal injection of d‐glucose (1 g·kg^−1^ of BW), and blood samples from a tail nick were taken at 0 (before injection), 15, 30, 45, 60, 90 and 120 min after glucose injection. Blood glucose levels were determined with a blood glucose meter (Sannuo, chang sha, China). The area under the blood concentration–time curve (AUC) of the GTT was calculated according to the following equation:

AUC = (blood glucose at 0 min + blood glucose at 15 min) × 7.5 + (blood glucose at 15 min + blood glucose at 30 min) × 7.5 + (blood glucose at 30 min + blood glucose at 45 min) × 7.5 + (blood glucose at 45 min + blood glucose at 60 min) × 7.5 + (blood glucose at 60 min + blood glucose at 90 min) × 15 + (blood glucose at 90 min + blood glucose at 120 min) × 15.

Three days after the GTT, an ITT was performed with insulin intraperitoneal injection (0.25 IU·kg^−1^ of body weight; Wanbang Biopharmaceuticals Co., Ltd., Shanghai, China) in mice after a 4‐h fast, followed by blood collection from a tail nick at 0 (before injection), 30, 60, 90 and 120 min after insulin injection. Blood glucose concentration was determined in the same way described above.

### Quantitative PCR analysis

To avoid acute effects of the last hypoxia session, the hypoxia exposure mice were allowed to rest for 48 h under normoxia with access to food and water. Subsequently, the mice were euthanized by cervical dislocation. The quadriceps femoris muscles were collected, cleaned and quick‐frozen in liquid nitrogen, and then stored at −80 °C.

Total RNA was isolated from ~ 50 mg of crushed muscle tissue using TRI reagent according to the manufacturer's instructions. Real‐time PCR was performed in an ABI 7500 Real‐time PCR System (Thermo Scientific, Inc., Waltham, MA, USA) using the SYBR Green Real‐time PCR Master Mix kit (Toyobo Co., Ltd, Osaka, Japan) with the previously synthesized cDNA (FSQ‐101; Toyobo Co., Ltd) as template in a 20 μL reaction volume. The following primers from Qiagen (Düsseldorf, Germany) were used as follows: glucose transporter type 4 (*Slc2a4*; QT01044946), 1,4‐α‐glucan branching enzyme 1 (*Gbe1*; QT00252924), phosphorylase kinase regulatory subunit α 1 (*Phka1*; QT00143514), hexokinase 2 (*Hk2*; QT00155582), muscular phosphofructokinase (*Pfkm*; QT00159754), mitochondrial uncoupling protein 3 (*Ucp3*; QT00115339), mitochondrial transcription factor A (*Tfam*; QT00154413), cytochrome *c* oxidase subunit 4 isoform 2 (*Cox4‐2*; QT00137844), estrogen‐related receptor α (*Esrr*α; QT00172998), nuclear respiratory factor 1 (*Nrf1*; QT01051820), peroxisome proliferator‐activated receptor α (*Ppar*α; QT00137984), and 18S ribosomal RNA (*Rn18s*; QT010036875). The *Rn18s* gene is a reliable internal control for comparative analyses of transcription under hypoxia 21, which is assessed using software (ABI 7500RT PCR). The difference in expression between control and experimental samples was calculated using the $2^{-\Delta \Delta C_{t}}$method, as described previously 22.

### Western blotting

Total proteins were isolated from 50 mg of whole hind limb muscles using T‐PER tissue protein extraction reagents (78510; Thermo Fisher Scientific, Inc.). Protein concentration was measured using the BCA protein assay kit (Pierce 23225; Thermo Fisher Scientific, Inc.). Total proteins (20 μg) were separated on Bolt 4–12% Bis‐Tris PlusGels (NW04125BOX; Thermo Fisher Scientific, Inc.) by electrophoresis, and the fractionated proteins were subsequently transferred to a nitrocellulose membrane using iBlot Gel Transfer Stacks Nitrocellulose (IB23001; Thermo Fisher Scientific, Inc.). The blots were probed using the following antibodies: AMP‐activated protein kinase α (AMPKα; SC‐74461; Santa Cruz Biotechnology, Inc., Dallas, TX, USA), Thr^172^‐phosphorylated (p)‐AMPKα (sc‐33524, Santa Cruz Biotechnology, Inc.), AKT (no. 4691; Cell Signaling Technology, Inc., Danvers, MA, USA), Ser^473^‐p‐AKT (#4060; Cell Signaling Technology, Inc.), glucose transporter 4 (GLUT4; ab64; Abcam, cambridge, England) and β‐actin (sc‐477778; Santa Cruz Biotechnology, Inc.). The density of protein bands was analyzed using Bio‐Rad imaging software (Bio‐Rad Laboratories, Hercules, CA, USA). The individual values were originally expressed as a ratio of a standard (β‐actin content) and then expressed as a fold change of the control group value.

### Plasma insulin

Plasma insulin concentration was assessed according to the manufacturer's instructions with a rat/mouse insulin ELISA kit (EMD Millipore, Temecula, CA, USA). Briefly, 10 μL of each plasma sample and 80 μL of detection antibody were added in the insulin antibody‐coated wells on the microplate. After a 2‐h incubation, the plate was washed, and enzyme solution was added at room temperature. After a 30‐min incubation, the plate was washed again. Following this, the substrate was added and the reaction was stopped after 15 min. The plate was read at 450 nm (Bio Tek Synergy H1, Bio Tek Instruments, Inc., winooski, VT, USA).

### Statistical analyses

All values are reported as the mean ± standard error (SE). Statistical calculations were performed using spss statistics v. 19 software (IBM Corp., Armonk, NY, USA). Data were analyzed using a two‐way ANOVA (strain × hypoxia). When a significant interaction effect was obtained, simple main effect analysis with the *post hoc* LSD test was performed to identify significant mean differences between groups. Statistical significance was set at *P* < 0.05.

## Results

### Confirmation of apelin KO mice

The genotypes of the mice from apelin homozygotes were determined by PCR using genomic DNA (Fig. S1).

### Effect of hypoxia exposure on glucose and insulin tolerance

**Figure 1:**
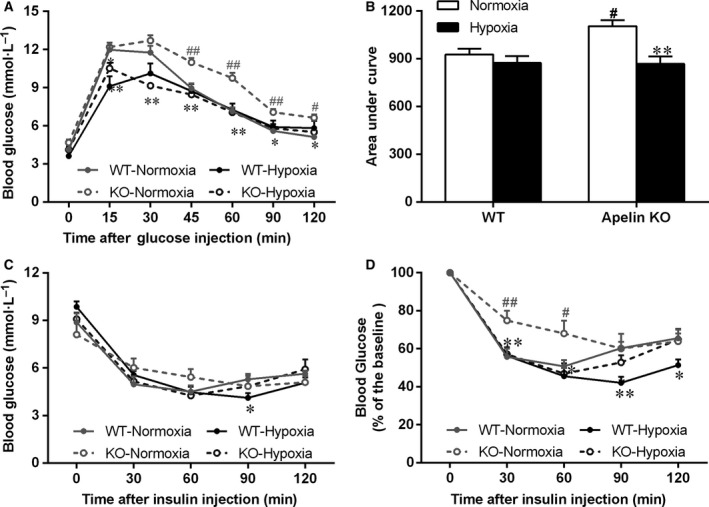
Glucose (1 g·kg^−1^) and insulin (0.25 IU·kg^−1^) tolerance tests for apelin KO and WT mice. (A) Glucose tolerance depicted as blood glucose *vs* time of post‐glucose injection. (B) Area under the curve of the blood glucose graph. (C) Insulin tolerance depicted as blood glucose *vs* time of post‐glucose injection. (D) Percentage of basal blood glucose. Values are displayed as the mean ± SE (*n* = 9 animals/group). *P < 0.05 and **P < 0.01, Hypoxia *vs* Normoxia; ^#^ *P* < 0.05 and ^##^ *P* < 0.01, apelin KO *vs* WT mice. Data were analyzed using two‐way ANOVA (strain × hypoxia).

During the GTT, the blood glucose levels were significantly higher in the KO–Normoxia group compared with the WT–Normoxia group, whereas the blood glucose levels were significantly lower in the KO–Hypoxia group compared with the KO–Normoxia group. No significant difference in blood glucose levels was found between apelin KO and WT mice after hypoxia exposure. The AUC values were consistent with the results of the GTT (Fig. 1A,B). These outcomes suggest that apelin KO mice had impaired glucose tolerance and that 4 weeks’ intermittent hypoxia was able to reverse the effects.

During the ITT, the blood glucose levels were significantly higher in the KO–Normoxia group compared with the WT–Normoxia group after insulin injection (Fig. S2). Reduced blood glucose levels were observed in the KO–Hypoxia group at the same time points when compared with the KO–Normoxia group, indicating hypoxia treatment might improve insulin sensitivity in apelin KO animals (Fig. 1C,D). As observed in the GTT results, apelin KO mice exhibited decreased insulin tolerance, which was improved by the 4 weeks’ intermittent hypoxia. In addition, there was no significant difference in glucose levels during the GTT and ITT tests between the WT–Normoxia and WT–Hypoxia groups, indicating the current hypoxia treatment did not affect the glucose and insulin tolerance in normal animals.

### Effect of hypoxia exposure on ${\dot{V}}_{O_{2}\max}$

**Figure 2:**
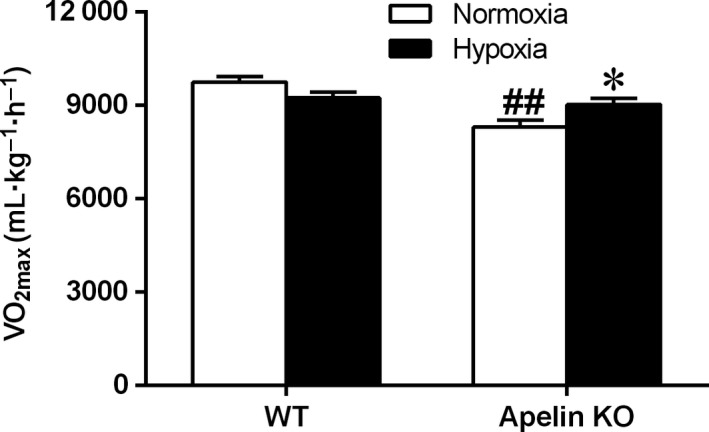
${\dot{V}}_{O_{2}\max}$ of WT and apelin KO mice after the 4 weeks’ intermittent hypoxia exposure. Values are displayed as the mean ± SE (*n* = 9 animals/group). *P < 0.05, Hypoxia *vs* Normoxia; ^##^ *P* < 0.01, apelin KO *vs* WT mice. Data were analyzed using two‐way ANOVA (strain × hypoxia).

Significantly decreased ${\dot{V}}_{O_{2}\max}$ was indicated in the KO–Normoxia group when compared to that of the WT–Normoxia group. However, the decreased ${\dot{V}}_{O_{2}\max}$ could be improved by hypoxia treatment (Fig. 2). There was no significant difference in ${\dot{V}}_{O_{2}\max}$ between the WT–Hypoxia and WT–Normoxia groups. These results indicated that the hypoxia exposure improved the decreased ${\dot{V}}_{O_{2}\max}$ in apelin KO mice but had no effect on normal WT mice.

### Effect of hypoxia exposure on mRNA and protein expression of muscular genes involved in glucose metabolism

**Figure 3:**
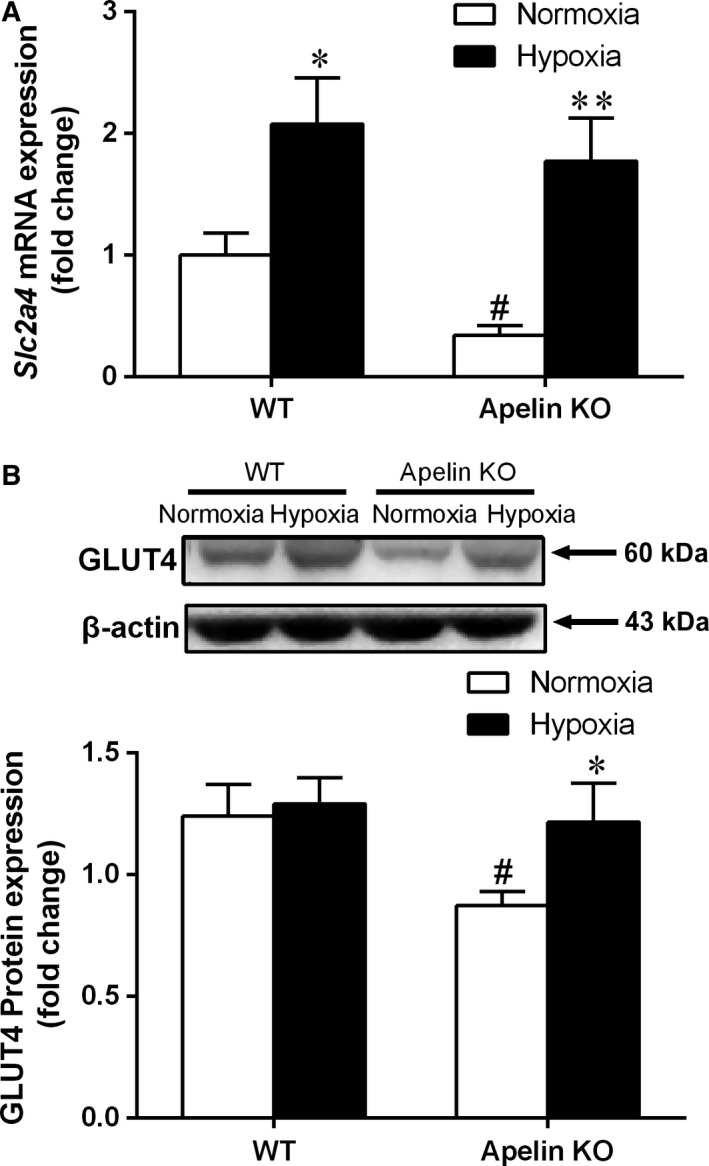
(A) *Slc2a4* mRNA and (B) GLUT4 protein expression levels in skeletal muscle of apelin KO and WT mice after 4 weeks’ intermittent hypoxia exposure. Values are displayed as the mean ± SE (*n* = 9 animals/group). *P < 0.05 and **P < 0.01, Hypoxia *vs* Normoxia; ^#^ *P* < 0.05, apelin KO *vs* WT mice. Data were analyzed using two‐way ANOVA (strain × hypoxia).

To investigate the role of skeletal muscle on glycemic control in apelin KO mice, we measured glucose metabolism variables in skeletal muscle. The expression of *Slc2a4*, which encodes GLUT4 protein 23 and is a key glucose transporter, was significantly reduced at both mRNA and protein levels in skeletal muscle from apelin KO mice when compared with WT mice. However, 4 weeks of hypoxia resulted in significantly increased mRNA and protein expression levels in the KO–Hypoxia group compared with the KO–Normoxia group (Fig. 3).

**Figure 4:**
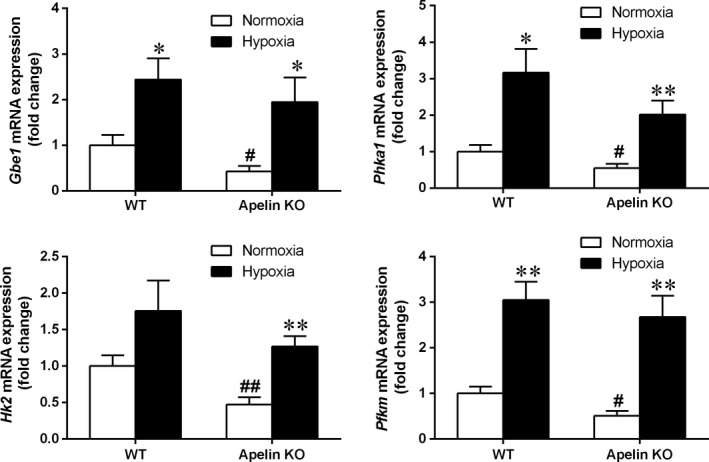
The mRNA expression levels of key genes of glycogen and glucose metabolism in skeletal muscle of apelin and WT mice after 4 weeks’ intermittent hypoxia exposure. Values are displayed as the mean ± SE (*n* = 9 animals/group). *P < 0.05 and **P < 0.01, Hypoxia *vs* Normoxia; ^#^ *P* < 0.05, ^##^ *P* < 0.01, apelin KO *vs* WT mice. Data were analyzed using two‐way ANOVA (strain × hypoxia).

*Gbe1* and *Phka1*, which encode glycogen branching enzyme 24 and phosphorylase b kinase 25, respectively, are the key enzymes of glycogen branching and breakdown, and *Hk2* and *Pfkm* encode hexokinase 2 26 and muscular phosphofructokinase 27, which are the key glycolytic enzymes. Without hypoxia exposure, apelin KO mice exhibited lower mRNA expression levels of these genes compared with WT mice, supporting our hypothesis of the functional influence of apelin KO on these genes. However, with hypoxia treatment both KO and WT mice expressed significantly elevated mRNA expression levels of these genes (Fig. 4), suggesting hypoxia treatment could elevate the expression of these genes, regardless of apelin KO.

### Effect of hypoxia exposure on mRNA expressions of muscular genes of fatty acid oxidation and mitochondrial metabolism

**Figure 5:**
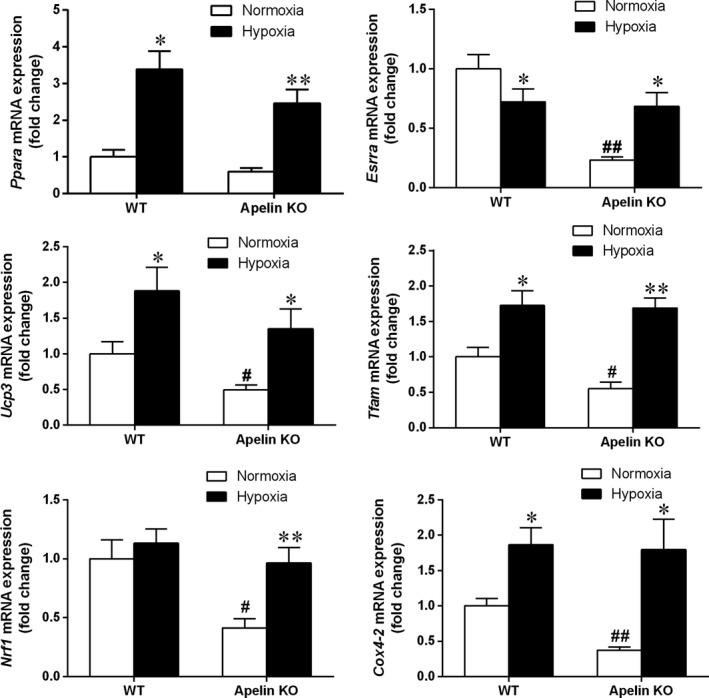
The mRNA expression levels of genes of fatty acid oxidation and mitochondrial metabolism in skeletal muscle of apelin and WT mice after 4 weeks’ intermittent hypoxia exposure. Values are displayed as the mean ± SE (*n* = 9 animals/group). *P < 0.05 and **P < 0.01, Hypoxia *vs* Normoxia; ^#^ *P* < 0.05 and ^##^ *P* < 0.01, apelin KO *vs* WT mice. Data were analyzed using two‐way ANOVA (strain × hypoxia).

In addition to the muscular genes measured in glucose metabolism, the mRNA expression levels of muscular genes of fatty acid oxidation and mitochondrial metabolism, including *Ppara*,* Ucp3*,* Esrra*,* Nrf1*,* Tfam* and *Cox4‐2*, were also investigated. Notably, peroxisome proliferator‐activated receptor α (PPARα; encoded by *Ppara*) promotes fatty acid oxidation 28 and *Ucp3* expression. *Ucp3* encodes uncoupling protein 3 (UCP3), a mitochondrial transporter protein, and plays a role in the modulation of tissue respiratory control 29. Furthermore, estrogen‐related receptor α (ERRα; encoded by *Esrra*) regulates mitochondrial biogenesis 30, oxidative phosphorylation 31 and fatty acid metabolism 32. Previous findings have also demonstrated that nuclear respiratory factor 1 (NRF1; encoded by *Nrf1*) increases the transcription of many genes required for mitochondrial respiratory function, including *Tfam* (encoding mitochondrial transcription factor A; TFAM) and *Cox4‐2* (cytochrome *c* oxidase subunit 4 isoform 2; COX4‐2) 33, 34. Like the results regarding the genes of glucose metabolism, apelin KO mice had lower mRNA expression levels of these measured genes (*Ppara*,* Ucp3*,* Esrra*,* Nrf1*,* Tfam* and *Cox4‐2*) compared with WT mice. However, with hypoxia treatment, the expression levels of these genes were significantly increased in both KO and WT mice. Interestingly, only the mRNA expression of *Esrra* in the WT–Hypoxia group was significantly lower than that of the WT–Normoxia, which differed to other observed genes such as *Ppara*,* Ucp3*,* Tfam* and *Cox4‐2* (Fig. 5).

### Effect of hypoxia exposure on plasma insulin level and the expression levels of p‐AMPK (Thr172) and p‐AKT (Ser‐473)

**Figure 6:**
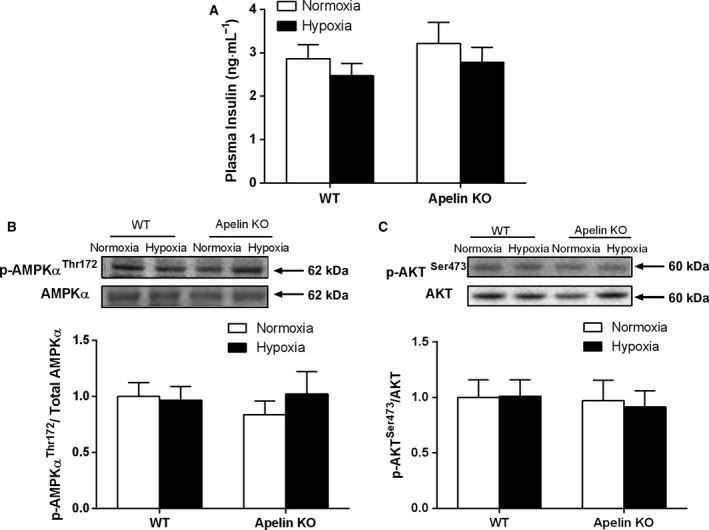
The levels of plasma insulin (A), p‐AMPK (Thr172) (B), and p‐AKT (Ser473) (C) in skeletal muscle of apelin and WT mice after 4 weeks’ intermittent hypoxia exposure. Values are displayed as the mean ± SE (*n* = 9 animals/group). Data were analyzed using two‐way ANOVA (strain × hypoxia).

To obtain more molecular evidence of the effects of apelin deficiency and hypoxia exposure on the expression of factors associated with muscular substrate metabolism, the protein expression levels of p‐AMPKα (Thr172) and p‐AKT (Ser‐473) in skeletal muscle, and plasma insulin levels were explored. However, there were no significant differences in the expression levels of p‐AMPKα (Thr172) and p‐AKT (Ser‐473) or plasma insulin between the KO–Normoxia and WT–Normoxia groups, between the KO–Hypoxia and KO–Normoxia groups, and between the WT–Hypoxia and WT–Normoxia groups, respectively (Fig. 6). The results suggested that these factors may not be involved directly in apelin and hypoxia regulations; the possible involvement of other mediators is currently unknown.

## Discussion

The findings of the current study revealed that apelin KO mice reduced the expression of factors associated with muscular substrate metabolism, impaired glucose and insulin tolerance, and decreased aerobic capacity; however, these negative manifestations were significantly reduced after 4 weeks’ intermittent hypoxia exposure. These results support the hypotheses of the present study. Moreover, proof was found that promoting muscular substrate metabolism after hypoxia exposure may contribute, at least in part, to the improvement of glucose tolerance and aerobic capacity in apelin KO mice.

Previous studies have demonstrated that dysregulation of apelin signaling was associated with pathological states, such as T2D and obesity 35. Apelin‐null mice have impaired glucose tolerance and insulin sensitivity 14, and lower exercise capacity compared with WT mice 17. In the present GTT, ITT and ${\dot{V}}_{O_{2}\max}$ test, significant decreases in glucose and insulin tolerance and ${\dot{V}}_{O_{2}\max}$ were observed in the apelin KO mice. These results supported the previous studies that apelin KO mice have decreased glucose tolerance and aerobic capacity compared with WT mice.

As the largest metabolic organ in the body and the most important target tissue of insulin, skeletal muscle may have a critical role in glucose disposal and ${\dot{V}}_{O_{2}\max}$ changes in apelin KO mice. GLUT4 is a key factor in controlling the transport of glucose into skeletal muscle 36. Its impaired expression and/or its disruption on the pathway may result in insulin resistance and glucose intolerance 37. To better understand the apelin KO‐related changes of muscular glucose transport and metabolism, we measured *Slc2a4* and its encoded protein, GLUT4, in the skeletal muscle of apelin KO mice. The results showed that apelin deficiency significantly reduced the mRNA expression of *Slc2a4* and protein expression of GLUT4. Moreover, apelin deficiency also down‐regulated the mRNA expression of *Gbe1* and *Phka1*, which encode important enzymes of glycogen branching and breakdown, and down‐regulated the mRNA expression of *Hk2* and *Pfkm*, which encode key enzymes of glycolysis. These data provided evidence of reduced glucose transport, glycogen and glucose metabolism in skeletal muscle of apelin KO mice, suggesting an apelin deficiency‐related diminishment of glucose uptake and utilization in skeletal muscle may contribute to the decreased glucose tolerance in apelin KO mice.

To identify the changes of mitochondrial biogenesis and fatty acid metabolism in skeletal muscle of mice with apelin deficiency, mRNA expression levels of several nuclear receptors, namely NRF1 (encoded by *Nrf1*), ERRα (encoded by *Esrra*) and PPARα (encoded by *Ppara*), and some target genes (*Tfam*,* Cox4‐2* and *Ucp3*), were studied. One of the pathophysiological characteristics of insulin resistance in skeletal muscle is the decreased rate of lipid oxidation and mitochondrial dysfunction 38. In this study, the expression levels of genes associated with the regulation of fatty acid oxidation and mitochondrial metabolism (*Ppara*,* Esrra*,* Ucp3*,* Nrf1*,* Tfam* and *Cox4‐2*) were all down‐regulated in skeletal muscle of apelin KO mice (Fig. 5). Taken together of the results from glucose metabolism, fatty acid metabolism and mitochondrial biogenesis in skeletal muscle, the decreased expression levels of these genes may be associated with reduced aerobic capacity in apelin KO mice.

Beneficial effects of hypoxia on diabetes, obesity and insulin sensitivity in people who live at higher altitudes 39, in obese subjects 6 and in diabetic rats 40 have been indicated in previous studies. It was reported that apelin regulates insulin sensitivity, stimulates glucose utilization and enhances brown adipogenesis in different tissues associated with diabetes 12, 41, indicating the apelin–APJ system as a novel therapeutic target for pharmacological intervention in treating diabetes. In order to further investigate the potential effects of 4 weeks’ intermittent normobaric hypoxia on apelin KO mice, we measured GTT, ITT, ${\dot{V}}_{O_{2}\max}$, protein expression of GLUT4, and the mRNA expression of genes involved in glucose, fatty acid and mitochondrial metabolism. The results indicated that 4 weeks’ exposure to normobaric hypoxia was able to reverse the decreased muscular substrate metabolism, glucose tolerance and aerobic capacity in apelin KO mice, and could improve these variables to the normal levels observed in the WT mice. The findings suggested that hypoxia exposure had antagonistic effects. Moreover, hypoxia may play a critical role in the energy homeostasis of skeletal muscle and provoke metabolic adaptation 42. Our results showed that the apelin KO–Hypoxia group exhibited higher expression levels of genes associated with muscular glucose metabolism and fat metabolism, and components involved in mitochondrial function compared with the KO–Normoxia group. These findings suggest that these genes may be important contributors in glucose tolerance and aerobic capacity in apelin KO mice.

To further research the potential molecular mechanism on regulating changes in muscular substrate metabolism in both WT and apelin KO mice under hypoxia, we measured the protein expression levels of p‐AMPKα (Thr172) and p‐AKT (Ser‐473) using western blotting, and plasma insulin concentration using an ELISA assay. However, apelin KO mice did not show any abnormal changes in plasma insulin or the protein expression levels of p‐AMPKα (Thr172) and p‐AKT (Ser‐473) compared with WT mice. This result was different from a previous study from Dray *et al*. 15, which reported that AMPK and AKT‐dependent signaling pathways may be involved in apelin regulation of glucose uptake in skeletal muscle of normal mice. The different experimental protocols may partially contribute to this discrepancy. In the present study, we used the apelin KO mice, while Dray *et al*. used acute intravenous injection of apelin in normal mice. Future research is required on this topic.

Hypoxia (13.3%) treatment did not significantly change the p‐AMPK‐to‐AMPK ratio of skeletal muscle in both WT and apelin KO mice in the present study. This finding is similar to our previous reports 43, 44, which did not observe any significant effects following 4 weeks of hypoxia treatment (11.2% O_2_) on AMPKα phosphorylation in skeletal muscles of WT, AMPKα2 KO and high‐fat diet‐induced obese mice. In all these studies, muscle tissue collection was performed at least 48 h after the last hypoxia treatment; however, whether this experimental protocol influenced AMPKα phosphorylation is currently unknown. In addition, the hypoxia treatment from the present study did not influence the levels of AKT phosphorylation (Ser473) in skeletal muscle or plasma insulin concentration in both apelin KO and WT mice. The results support the theory that the phosphoinositide 3‐kinase/AKT signaling pathway may not participate in the responses to hypoxia 45. Based on these outcomes, the complete mechanism of hypoxia exposure on improving muscular substrate metabolism in skeletal muscle of apelin KO mice requires further study.

It is important to acknowledge the limitations of our study. In this study, we only focused on whether hypoxia exposure could reverse the impaired muscular metabolism, glucose tolerance and aerobic capacity in apelin KO mice. Hypoxia‐inducible factor 1 (HIF1) has been identified as a master regulator for the expression of genes involved in the hypoxia responses, such as genes coding for glucose transporters and glycolytic enzymes 46. It is possible that some changes observed in the present study would be mediated via HIF1. However, the tissues were collected after a 48 h normoxia recovery of the last hypoxia session. Whether HIF1 or other factors are involved in the hypoxia regulation cannot be explained well in the current study design. Therefore, future research on the role of HIF1 in the metabolism of the apelin KO mouse is needed.

In conclusion, apelin KO mice have impaired glucose tolerance and reduced exercise capacity compared with WT mice. Moreover, the present findings revealed that there were decreased expression levels of genes associated with substrate metabolism in skeletal muscle. To the best of our knowledge, this study demonstrated for the first time that 4 weeks’ intermittent hypoxia was able to reverse decreased muscular substrate metabolism, glucose tolerance and aerobic capacity in apelin KO mice. The findings provide novel molecular evidence that hypoxia intervention can correct metabolic conditions caused by genetic defects.

## Conflict of interest

The authors declare no conflict of interest.

## Author contributions

SH and YZ designed the research; SH and JL performed the experiments. SH analyzed the data and visualization. YZ contributed reagents/materials and YZ and JW wrote and revised the paper.

## Supporting information


**Fig. S1.** PCR analysis of genomic DNA of WT and apelin KO mice.

**Fig. S2.** Area under the curve of the blood glucose graph (ITT).
