# Supplementary figures and images for: Hypoxia exposure alleviates impaired muscular metabolism, glucose tolerance, and aerobic capacity in apelin‐knockout mice
Source: FEBS Open Bio. 2019 Jan 23;9(3):498–509. doi: 10.1002/2211-5463.12587 (PMC6396165; doi:10.1002/2211-5463.12587)

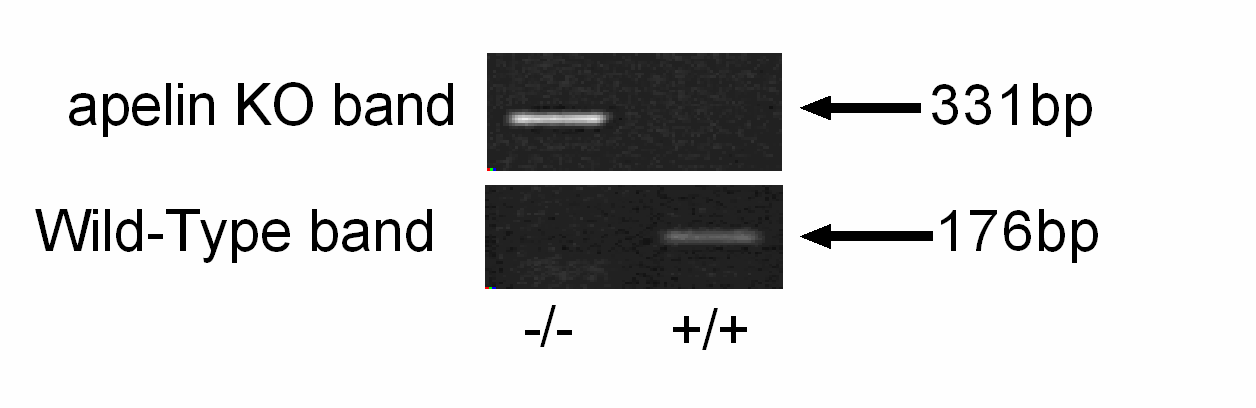

Supplement: Supplementary file 1 — Fig. S1. PCR analysis of genomic DNA of WT and apelin KO mice. [file FEB4-9-498-s001.tif]

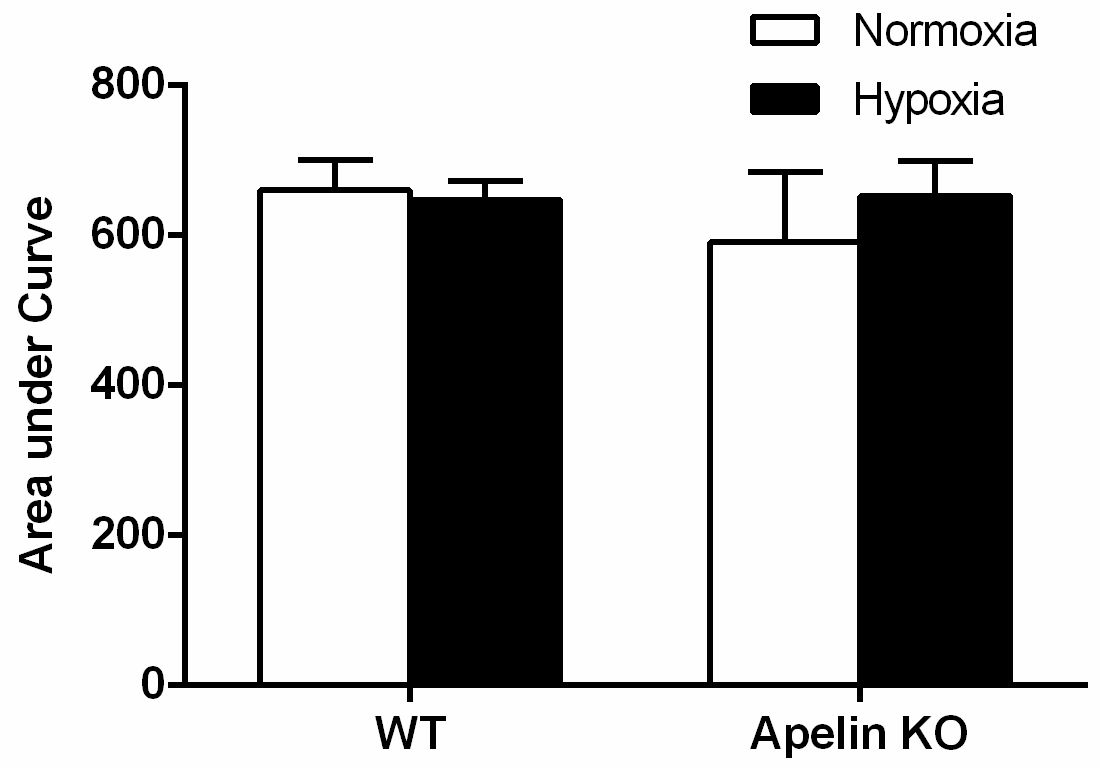

Supplement: Supplementary file 2 — Fig. S2. Area under the curve of the blood glucose graph (ITT). [file FEB4-9-498-s002.tif]
